# Interpersonal Trust and Quality-of-Life: A Cross-Sectional Study in Japan

**DOI:** 10.1371/journal.pone.0003985

**Published:** 2008-12-19

**Authors:** Yasuharu Tokuda, Masamine Jimba, Haruo Yanai, Seiji Fujii, Takashi Inoguchi

**Affiliations:** 1 Center for Clinical Epidemiology, St Luke's Life Science Institute, St Luke's International Hospital, Tokyo, Japan; 2 Department of International Community Health, Graduate School of Medicine, The University of Tokyo, Tokyo, Japan; 3 Department of Biostatistics, St. Luke's College of Nursing, Tokyo, Japan; 4 Graduate School of Political Science, Chuo University, Tokyo, Japan; University of Otago, New Zealand

## Abstract

**Background:**

There is growing interest in psychosocial factors with positive attitudes, such as interpersonal trust, as determinants for Quality-of-life (QOL) or subjective well-being. Despite their longevity, Japanese people report a relatively poor subjective well-being, as well as lower interpersonal trust. Our aim in this study was to evaluate the possible association between interpersonal trust and QOL among Japanese people.

**Methodology and Principal Findings:**

Based on the cross-sectional data for Japanese adults (2008), we analyzed the relationship between interpersonal trust and each of four domains of the WHOQOL-BREF. Interpersonal trust was assessed using three scales for trust in people, in human fairness and in human nature. In a total of 1000 participants (mean age: 45 years; 49% women), greater trust was recognized among women (vs. men), those aged 60–69 (vs. 20–29), or the high-income group (vs. low-income). Each of three trust scales was positively correlated with all domains of QOL. Multiple linear-regression models were constructed for each of QOL and the principal component score of the trust scales, adjusted for age, gender, area size of residence, income, education, and occupation. For all QOL domains, interpersonal trust was significantly and positively associated with better QOL with p<0.001 for all four domains including physical, psychological, social, and environmental QOL. Other factors associated with QOL included gender, age class, area size of residence, and income. Education and occupation were not associated with QOL.

**Conclusions and Significance:**

Greater interpersonal trust is strongly associated with a better QOL among Japanese adults. If a causal relationship is demonstrated in a controlled interventional study, social and political measures should be advocated to increase interpersonal trust for achieving better QOL.

## Introduction

Quality of life (QOL), or subjective well-being, is a critical aspect of individual welfare and is a worthy goal for societies. In addition to health-related common risk factors, such as genetics, demographics, life-styles, and environmental factors, a growing body of research shows that multiple socioeconomic factors are also considered as important determinants for QOL.

For example, previous studies have demonstrated relationships between income and subjective well-being. High-income provides a better QOL when it lifts them out of abject poverty and into the middle class, but it does little to increase QOL thereafter [1]. Next, work provides income as well as extra-meaning of life to individuals through a feeling of contributing to society. Unemployment reduces income, but also it reduces the level of QOL. In addition, the level of control that individual workers have over their jobs is also an important issue. For instance, among British civil servants in all hierarchical ranks, those who perform the most uncontrollable and routine work are at the highest risk for poor health and premature mortality [2].

There is growing interest in psychosocial factors with positive attitudes, such as trust, optimism, and sociability, as determinants for subjective well-being [3], [4], [5], [6]. Among these factors, interpersonal trust is now considered as an important positive predictor of subjective well-being [7], [8]. Trust is a belief that the sincerity or the good will of others can be generally relied upon [9]. Development of the capacity to trust others is essential for developing an integrated personality and successful social adjustment [10].

In contrast, negative attitudes, such as mistrust, hostility, suspiciousness, and cynicism, are related to poor psychological well-being [11]. Among these negative attitudes, mistrust is the cognitive habit of interpreting the intentions and behavior of others as dishonest, unsupportive, and self-seeking. The central cognitive component of mistrust is suspicion of others based on a belief that they are looking out for their own good and they will even victimize you in pursuit of their own personal goals [12].

Mistrusting people believe it is safer to keep their distance from others. Mistrust can also hinder the development, maintenance, and the use of social support networks. Further, mistrusting individuals are less likely to seek social support when in need, may be uncomfortable with any support, and may even reject offers of support. By establishing this vicious cycle, mistrusting individuals can elicit hostile responses from others and unfriendly conditions that may justify their beliefs. Moreover, they can be easy targets of exploitation and crime due to little reciprocity and no mutual assistance among social networks. Mistrust thus causes poor well-being and can even develop into paranoia with a higher risk for suicide [13].

There are warning indications of trends within industrialized countries with regard to social disconnection and poor subjective well-being [14], [15]. For instance, Japan is one of the richest countries, and the degree of income equality has been relatively stable based on international comparative data [16]. Furthermore, the Japanese people have the highest life expectancy in the world [17]. However, according to the international values survey, the Japanese are poor regarding subjective well-being, or “the most unhappy”, among the industrialized countries [18]. Furthermore, based on our previous survey, the Japanese report relatively lower levels of interpersonal trust compared with other countries [19].

Despite the importance of investigating the association between trust and QOL, few studies have evaluated this relationship in Japan and it is unclear whether interpersonal trust is related to the QOL of the Japanese people. Thus, we aimed to evaluate the association between interpersonal trust and the QOL among Japanese adults. If this association could be confirmed in this population, controlled interventional studies should be conducted to confirm its causal relationship and then a policy could be instituted to enhance people's QOL in Japan. Furthermore, these findings might be generalizable to populations in other countries.

## Methods

### Study participants

Ethics approval was obtained from the University of Tokyo, Graduate School of Medicine prior to beginning the study. Verbal informed consent was obtained from all participants because of the limited time for survey interviewing and waiver of written consent was authorized by the ethics committees. We classified all municipalities in Japan into 10 regions, including Hokkaido, Tohoku, Kanto, Tokai, Chubu, Hokuriku, Kinki, Chugoku, Shikoku and Kyushu. In each region, municipalities were stratified into four categories corresponding to their population sizes, as follows; 1) 12 metropolises: Sapporo, Sendai, Chiba, Tokyo (metropolitan area), Yokohama, Kawasaki, Nagoya, Osaka, Kobe, Hiroshima, Kita-Kyushu and Fukuoka; 2) cities with a population of 100,000 or greater, 3) cities with a population less than 100,000, and 4) towns or villages.

All municipalities in Japan were stratified into 100 blocks. Within each block, primary sampling units (census tracts) were randomly chosen through probability proportionate to the sampling size, similar to the national census data of population distributions for 20–69 years old in 2005. Eligible household individuals were randomly chosen from each resident registration ledger of the census tracts. Within a unit identified for sampling, the households were selected randomly using the Right Hand Walk rule, in which households were contacted in clusters around the selected starting points. From the first household contacted, two households were skipped and the next one contacted. If we would have interviewed the first eligible member who was available at the time of the survey, this could lead to a non-random sample, since it could lead to an over-representation of women, as women are easier to interview and are more likely to be available. To avoid this problem, we used the Kish Grid, a method of selecting eligible respondents randomly from within a household using a random number table. Using this method, we did not stop the sampling until we obtained a sample size of 1000 persons.

### Data collection

Face-to-face interviews were used to administer structured questionnaires between January 10 and 27, 2008. Data collection included demographics, marital status, socioeconomic factors (income, education, and occupation), health-related quality of life (QRQOL), and interpersonal trust, in addition to information on political, environmental and social issues, which were related to the Asia Barometer Survey [19].

Age was categorized into five groups of 20–29, 30–39, 40–49, 50–59, and 60–69 years. Categories of marital status included; married (including unmarried but partnered) or others (single, divorced, separated, or widowed). Annual household income was used as a variable of income. Two income cutoff points of 5 and 8 million Japanese Yen (JY) were used to generate three income categories (Note: the average exchange rate to one US dollar in Jan 2008 was about 100 JY).

For educational attainment, the low-education group included participants who had completed primary school or junior high school. The mid-education group included participants who had completed high school. The high-education group included participants who had completed technical school, college, university or graduate school.

For occupational status, four categorical levels were used, including self-employed, homemaker, employed, or unemployed. The self-employed group included: 1) self-employed in agriculture, forestry or fisheries; 2) business owner in mining or manufacturing industry of an organization with up to 30 employees; 3) vendor or street trader; 4) business owner or manager of an organization; and, 5) self-employed professional. The employed group included: 1) senior manager; 2) employed professional or specialist; 3) clerical worker; 4) sales; 5) manual worker; 6) driver; and, 7) other worker. The unemployed group included: 1) student; 2) retired; and, 3) the unemployed.

The QOL was assessed using the Japanese version of the WHOQOL-BREF, which is the brief version of the WHOQOL-100. One item from each of the 24 facets contained in the WHOQOL-100 was included into this version to obtain a broad and comprehensive assessment. In addition, two items from the overall quality of life and general health facet were included. The WHOQOL-BREF contains a total of 26 items assessing four domains consisting of physical, psychological, social and environmental QOL. We excluded a single item regarding sexual satisfaction because we thought this item was considered likely to cause an emotional response in interviewees, and thus our instrument contained a total of 25 items (see Appendix S1). For comparing the scores between the domains, the WHOQOL-BREF scores were transformed into scores from 0–100 with the lowest score of zero and the highest score of 100. The reliability and validity of this instrument were confirmed previously [20].

For measuring interpersonal trust, we utilized the widely-used three items related to trust in people, human fairness and human nature [21], [22], [23]. For trust in people, we asked: “would you say that 1) most people can be trusted; or do you think 2) you can't be too careful in dealing with people?” By using the scale printed on a card, participants were required to choose one from a total of 11 natural numbers between 0–10. Choosing the first sentence in the highest agreement was considered to have a score of 10 (greatest trust), while the second sentence in the highest agreement had a score of zero (lowest trust).

For trust in human fairness, we asked: “do you think that 1) most people would try to be fair; or do you think that 2) they would try to take advantage of you if they got the chance. For trust in human nature, we asked: “Would you say that 1) most of the time people try to be helpful; or that 2) they are mostly looking out for themselves?” The choice of responses was similar to the above item and thus the higher the score, the greater the trust.

### Statistical analysis

Descriptive statistics were calculated and presented as the mean with standard deviation or count number with proportion where appropriate. Mean scores in interpersonal trust scales and in the QOL domains were calculated for each sociodemographic group. Mean scores between-groups were compared using ANOVA with pairwise comparisons based on Tukey's method. Correlation coefficients between the QOL domains and trust scales and among trust scales were calculated using Pearson's correlation coefficients.

Reliability and validity was examined for the WHOQOL-BREF. As a reliability measure, Cronbach's alpha was estimated for each domain. A multiple linear regression model was constructed for the combined general facet items (overall health plus overall QOL) as a dependent variable and the four domains as covariates, and R-square and standardized beta coefficients were estimated as a validity measure.

Principal component analysis (PCA) was employed to the three trust scales for yielding the principal component score (interpersonal trust score). In PCA, a set of variables is transformed into some linear combinations of the original variables by assigning weights to each variable so that the resulting composite variables as a set may have maximum variance under the restrictions that different linear composites are orthogonal to each other. The first PCA score attains the maximum variance among the linear combination of the three scales.

We then considered the following multiple regression models:
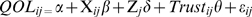
(1)where *QOL*_ij_ measured the QOL for the individual *i* living in the area *j*, X_ij_ was a set of participants' characteristics, Z*_j_* is a set of regional variables, *Trust*_ij_ was the first principal component score (interpersonal trust score) described above, and ε_ij_ was the error term. In the current study, the parameter of interest was *θ* adjusted for X_ij_ and Z*_j_,* since we aimed to examine possible association between *Trust*_ij_ and *QOL*_ij_. Standard errors of regression coefficients were estimated for each QOL domains by bootstrapping since the equations included the generated regressor (interpersonal trust score) from PCA. The coefficients of >zero indicated a positive relation to each QOL domain.

Finally, a structural equation modeling was constructed for examining the relationship between interpersonal trust and QOL as well as for assessing the magnitude of effect sizes for interrelationships among associated variables. Latent variables for three trust scales and for four QOL domains (trust and overall QOL, respectively) were constructed and path coefficients were estimated using the maximum likelihood method. For testing possible differences of the coefficients in the path of trust and overall QOL between both genders and between five age groups, the simultaneous multi-group analysis was conducted using equality restriction on these coefficients. The model was selected based on the Akaike's Information criterion (AIC), with a lower AIC indicating a better model. All statistical analyses were performed using SPSS 15.0J (SPSS Japan, Tokyo, Japan). Two-tailed p-values <0.05 were considered statistically significant.

## Results

Table 1 presents the sociodemographic characteristics of the participants. The mean age was 45 years with a standard deviation of 14 and women comprised 49%. There were 778 (78%) participants who were married. The highest number of participants (340, 34%) lived in the Kanto region. Regarding socioeconomic status, 328 (33%) reported an annual household income less than 5 million JY; 74 (7%) reported an attained education of junior high school or lower.

**Table 1:** Sociodemographics of participants.

Demographic	Subcategory	Participant (N = 1000)	
		n	%
Gender	men	505	51%
	women	495	49%
Age	20–29	191	19%
	30–39	215	22%
	40–49	189	19%
	50–59	212	21%
	60–69	193	19%
Region	Hokkaido/Tohoku	120	12%
	Kanto	340	34%
	Chubu	180	18%
	Kinki	160	16%
	Chugoku/Shikoku	90	9%
	Kyushu	110	11%
Area of residence	12 major cities	250	25%
	Cities with population > = 100k	410	41%
	Cities with population <100k	200	20%
	Rural areas	140	14%
Annual household income, JY	<5 million	328	33%
	> = 5 million & <8 million	259	26%
	> = 8 million	166	17%
	N/A	247	25%
Educational attainment	Junior high school or lower	74	7%
	High school	430	43%
	College or higher	493	49%
	N/A	3	1%
Occupation	Self-employed	134	13%
	Homemaker	164	16%
	Employed	587	59%
	Unemployed	275	28%
	N/A	4	1%

JY = Japanese Yen; N/A = data not available.

Table 2 presents the mean scores of the three trust scales by sociodemographic factors. Based on the between-group comparisons, women were more likely to report a greater trust in all three scales than men. Compared to other age groups, persons aged 60 years or older reported a greater trust in human fairness and nature, while those 20–29 years old reported a lower trust in human nature. There was no significant difference of these trust scales by area size of residence. Compared to other income groups, persons with ≥8 million JY reported a greater trust in people and human fairness. There was no significant difference in these trust scales by educational attainment. For occupational status, compared to the employed groups, homemakers reported a greater trust in human nature.

**Table 2:** Mean scores in interpersonal trust by sociodemographics.

Sociodemographic	Subcategory	Trust in people		Trust in human fairness		Trust in human nature	
		Mean	SD	Mean	SD	Mean	SD
Gender	men	5.3	2.1	5.7	1.7	4.8	1.8
	women	5.6	2.1	6.1	1.8	5.3	1.9
	t-statistic, P-value	2.206	0.028	3.675	<0.001	3.931	<0.001
Age	20–29	5.2	2.2	5.7	1.8	4.4*	1.7
	30–39	5.2	2.1	5.7	1.6	4.9	1.8
	40–49	5.5	1.9	5.7	1.5	5.1	1.6
	50–59	5.5	2.3	6.1	1.9	5.2	2.1
	60–69	5.6	2.2	6.3*	1.9	5.7*	1.9
	F-statistic, P-value	1.681	0.152	4.411	0.002	11.825	<0.001
Area size of residence	12 major cities	5.3	2.1	6.0	1.7	5.0	1.8
	Cities with population > = 100k	5.6	2.2	6.0	1.8	5.2	1.9
	Cities with population <100k	5.4	2.1	5.7	1.7	4.9	1.9
	Rural areas	5.2	2.0	5.9	1.8	5.0	1.8
	F-statistic, P-value	1.381	0.247	0.839	0.473	1.707	0.164
Annual household income, JY	<5 million	5.1	2.1	5.7	2.0	5.0	2.0
	> = 5 million & <8 million	5.5	2.2	5.9	1.6	5.1	1.8
	> = 8 million	6.1*	1.9	6.3*	1.5	5.3	1.6
	F-statistic, P-value	11.948	<0.001	6.620	0.001	1.982	0.138
Educational attainment	Junior high school or lower	5.0	2.2	5.9	1.9	5.2	2.2
	High school	5.4	2.1	5.9	1.9	5.1	1.8
	College or higher	5.5	2.1	5.9	1.6	5.0	1.8
	F-statistic, P-value	1.978	0.139	0.080	0.923	0.283	0.754
Occupation	Self-employed	5.4	2.4	6.0	1.9	5.2	1.7
	Homemaker	5.5	2.3	6.2	1.8	5.5*	1.9
	Employed	5.4	2.0	5.8	1.7	4.9*	1.9
	Unemployed	5.2	2.2	5.7	2.0	4.9	1.8
	F-statistic, P-value	0.337	0.798	1.936	0.122	3.478	0.016

* indicates a significant difference based on Tukey pairwise comparisons.

Cronbach's alpha of QOL domains was 0.74 for physical QOL, 0.73 for psychological QOL, 0.63 for social QOL, and 0.72 for environmental QOL. Multiple linear regression for the QOL domains with the overall health plus overall QOL showed an R-square value of 0.61; standardized beta coefficients were 0.34 (p<0.001) for physical, 0.21 (p<0.001) for psychological, 0.08 (p = 0.01) for social, and 0.10 (p = 0.004) for environmental QOL.

Table 3 shows the mean scores in QOL domains by sociodemographics. Based on the between-group comparisons, women had a higher social QOL than men. Regarding sub-groupings by 10-year age increments, a greater environmental QOL was noted among persons aged 60–69 years, while a lower environmental QOL was recognized among those aged 40–49. Persons living in cities with a population ≥100,000 had a greater environmental QOL but persons living in major cities had a lower environmental QOL.

**Table 3:** Mean scores in QOL domains by sociodemographics.

Sociodemographic	Subcategory	Physical (N = 989)		Psychological (N = 973)		Social (N = 987)		Environmental (N = 930)	
		Mean	SD	Mean	SD	Mean	SD	Mean	SD
Gender	men	69.4	15.1	55.1	14.8	65.2	16.0	59.3	13.5
	women	70.4	14.2	54.5	14.9	69.0	13.8	60.2	12.3
	t-statistic, P-value	0.989	0.323	0.670	0.503	3.954	<0.001	−0.996	0.320
Age	20–29	71.7	14.3	55.3	16.6	67.8	16.6	60.0	13.1
	30–39	69.4	16.4	56.3	16.0	67.9	14.0	58.6	13.1
	40–49	69.8	13.7	53.6	14.0	65.5	15.8	58.1*	13.2
	50–59	68.5	14.7	53.0	14.5	65.2	15.2	60.3	13.0
	60–69	70.2	13.7	55.9	12.6	68.9	13.4	61.9*	11.8
	F-statistic, P-value	1.319	0.261	1.932	0.103	2.316	0.056	2.442	0.045
Area size of residence	12 major cities	69.0	14.1	53.4	13.4	65.7	13.7	57.8*	12.0
	Cities with population > = 100k	70.8	14.6	55.8	14.5	67.6	14.2	61.1*	13.0
	Cities with population <100k	70.4	15.2	56.1	15.8	67.9	17.4	60.7	13.3
	Rural areas	68.1	14.9	52.4	16.6	66.7	16.3	57.9	13.2
	F-statistic, P-value	1.644	0.178	2.943	0.032	1.100	0.348	4.577	0.003
Annual household income, JY	<5 million	68.2*	15.8	54.3	14.8	66.2	15.5	57.5	13.6
	> = 5 million & <8 million	70.3	14.0	54.8	14.1	66.8	15.8	59.2	12.4
	> = 8 million	72.4*	13.3	57.6	15.2	69.4	14.1	63.7*	12.1
	F-statistic, P-value	4.683	0.010	2.916	0.055	2.483	0.084	12.335	<0.001
Educational attainment	Junior high school or lower	65.4*	14.3	51.1*	12.8	64.7	14.4	58.2	10.4
	High school	69.9	13.3	54.2	14.3	66.6	14.7	58.7	12.8
	College or higher	70.5	15.6	55.9*	15.3	67.8	15.5	60.9	13.2
	F-statistic, P-value	3.999	0.019	4.051	0.018	1.611	0.200	3.499	0.031
Occupation	Self-employed	69.5	13.1	55.1	13.9	67.2	13.1	60.2	11.3
	Homemaker	71.9	13.2	55.6	14.1	69.7	13.2	61.5	12.7
	Employed	69.6	15.1	54.7	15.1	66.5	15.6	59.0	13.3
	Unemployed	69.4	15.7	54.0	16.2	66.1	16.5	60.8	12.9
	F-statistic, P-value	1.145	0.330	0.303	0.824	2.042	0.106	1.799	0.146

* indicates a significant difference based on Tukey pairwise comparisons.

For annual household income, persons with an income <5 million JY had a lower physical QOL, whereas those with an income ≥8 million had a greater physical and environmental QOL. Similarly, for educational attainment, persons with junior high school or lower had a lower physical and psychological QOL. There was no significant difference in all QOL domains by occupation.

Table 4 presents the correlation coefficients between QOL and trust scales and also among trust scales. Moderate positive correlations were present between all domains of QOL and all three trust scales and high positive correlations were recognized among the trust scales. Based on the principal component analysis performed for these trust scales, a single factor with an eigenvalue of 1.76 and variance proportion of 59% was retained (interpersonal trust scale) and eigenvalues of no other principal components exceeded unity. The principal component loadings for the principal component were 0.76 for trust in people, 0.83 for trust in human fairness, and 0.71 for trust in human nature.

**Table 4:** Correlation between QOL and trust scales and among trust scales.

Domains of HRQOL	Trust in people		Trust in human fairness		Trust in human nature	
	r	P-value	r	P-value	r	P-value
Physical (N = 989)	0.135	<0.001	0.154	<0.001	0.093	0.003
Psychological (N = 973)	0.137	<0.001	0.191	<0.001	0.149	<0.001
Social (N = 987)	0.179	<0.001	0.228	<0.001	0.169	<0.001
Environmental (N = 930)	0.136	<0.001	0.221	<0.001	0.138	<0.001
Trust in people	-	-	0.458	<0.001	0.265	<0.001
Trust in human fairness	-	-	-	-	0.396	<0.001
Trust in human nature	-	-	-	-	-	-

HRQOL = health-related quality of life; r = Pearson's correlation coefficient.

Table 5 shows the results of multiple linear-regressions for QOL domains of sociodemographics and interpersonal trust. In these adjusted analyses, interpersonal trust was significantly and positively associated with all four domains of QOL. Higher interpersonal trust was related to the greater scores in all four QOL domains.

**Table 5:** Multiple linear-regressions for QOL dimensions of sociodemographics and interpersonal trust.

Covariate	Subcategory	Physical			Psychological			Social			Environmental		
		coefficient	SE	P-value	coefficient	SE	P-value	coefficient	SE	P-value	coefficient	SE	P-value
Gender	Men	reference			reference			reference			reference		
	Women	−0.48	1.25	0.70	−1.97	1.24	0.11	4.13	1.20	<0.001	−0.63	1.15	0.57
Age	20–29	reference			reference			reference			reference		
	30–39	−2.31	1.78	0.19	0.53	1.91	0.76	−0.42	1.80	0.81	−1.68	1.61	0.28
	40–49	−3.87	1.78	0.04	−3.39	1.96	0.07	−5.36	1.97	<0.001	−2.93	1.64	0.07
	50–59	−6.10	1.86	<0.001	−5.82	1.94	<0.001	−5.99	1.92	<0.001	−2.50	1.64	0.13
	60–69	−3.00	1.85	0.12	−0.94	1.93	0.63	−1.39	1.89	0.48	0.97	1.70	0.58
Area size of residence	12 major cities	reference			reference			reference			reference		
	Cities with population > = 100k	1.10	1.29	0.40	1.48	1.23	0.26	1.40	1.23	0.30	2.81	1.11	0.02
	Cities with population <100k	1.51	1.60	0.33	3.28	1.62	0.04	2.90	1.73	0.07	4.12	1.40	<0.001
	Rural areas	−0.68	1.80	0.72	0.28	1.86	0.88	2.81	1.92	0.14	0.88	1.69	0.60
Annual household income, JY	<5 million	reference			reference			reference			reference		
	> = 5 million & <8 million	2.49	1.29	0.05	0.60	1.30	0.64	0.51	1.32	0.70	1.97	1.12	0.09
	> = 8 million	4.40	1.47	<0.001	3.35	1.45	0.03	2.53	1.51	0.10	5.89	1.32	<0.001
Educational attainment	Junior high school or lower	reference			reference			reference			reference		
	High school	3.09	2.30	0.17	1.64	2.03	0.46	2.73	2.18	0.23	1.60	1.76	0.43
	College or higher	2.82	2.40	0.22	2.71	2.10	0.23	4.19	2.21	0.07	2.88	1.84	0.16
Occupation	Self-employed	reference			reference			reference			reference		
	Homemaker	1.01	2.08	0.65	−0.08	2.16	0.97	−3.03	2.18	0.19	2.05	2.03	0.32
	Employed	−1.56	1.58	0.38	−1.46	1.79	0.41	−2.87	1.75	0.12	−0.59	1.52	0.72
	Unemployed	0.71	2.44	0.78	−0.78	2.35	0.76	−2.55	2.63	0.33	1.95	2.19	0.40
Interpersonal trust	(PCS based on the trust scales)	2.24	0.57	<0.001	2.94	0.59	<0.001	3.50	0.62	<0.001	2.51	0.56	<0.001

QOL = quality of life; JY = Japanese Yen; PCS = principal component score. SE = standard error.

For other variables associated with QOL domains, including gender, age, area size of residence and income, women had a greater social QOL than men. Compared to persons aged 20–29, those aged 40–49 had a lower physical and social QOL; those aged 50–59 had a lower physical, psychological and social QOL. Compared to persons living in 12 major cities, those living in cities with population ≥100,000 had a greater environmental QOL; those living in cities with population <100,000 had a greater psychological and environmental QOL. Compared to persons with an income <5 million JY, those with income ≥8 million had greater physical, psychological and environmental QOL. There was no significant difference in all QOL domains by educational attainment and occupation.

**Figure 1:**
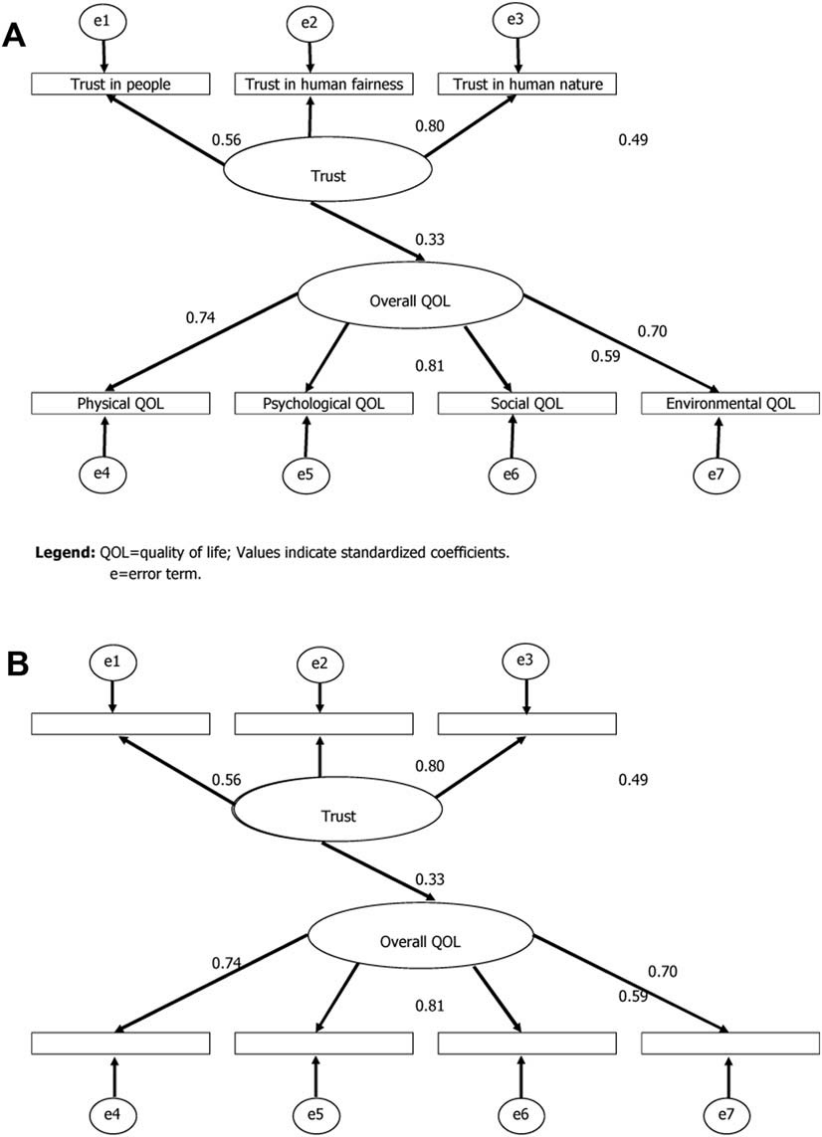
QOL = quality of life; Values indicate standardized coefficients. e = error term.

Figure 1 presents the structural equation model for interpersonal trust and QOL. Latent variables of trust and overall QOL were linked with the significant path coefficient (0.33, p<0.001). Among the QOL, the psychological domain contributed most to overall QOL, while the social domain contributed the least. For trust scales, trust in human fairness contributed most in the latent scale of trust, while trust in human nature contributed the least.

Based on the simultaneous multi-group analysis with equality restriction on these path coefficients by both genders and by five age groups, the models with equality restriction resulted in the better fit as compared with the model without the equality condition, because the AIC of the models by genders were: 87.1 for the model without the equality restriction and 85.8 for the model with the equality restriction. In addition, the AIC of the models by age groups were 252.3 for the model without the equality restriction and 250.6 for the model with the equality restriction.

## Discussion

Our study presents cross-sectional evidence of a significant association between interpersonal trust and better QOL in the Japanese people, after adjustment for age, gender, regions, area size of residence, income, education, and occupation. People with a greater sense of interpersonal trust are more likely to report that they have greater QOL in all domains, including physical, psychological, and environmental QOL, than people with lower trust.

These results are consistent with previous studies which have shown that a higher level of interpersonal trust is associated with better individual-level health status, including better health satisfaction and longer healthy longevity in an elderly population [7], lower mortality [24], better self-rated health in a Swedish bilingual community [25], better health in countries with high levels of social capital [26], and better physical and emotional health in Russians [27].

Based on the results of our study, we suggest several implications for further studies and public health policy-making. Policies to improve social skills, interpersonal ties, and social support to spread positive interpersonal trust might be important for improving global QOL. However, before developing a formal strategy to emphasize the acquisition of positive interpersonal trust along with the elimination of interpersonal mistrust, we need evidence showing a causal pathway from greater trust to better QOL and the significance and magnitude of this pathway can be evaluated in the context of a controlled interventional study. Regional pilot trials using a community randomized design may be optimal as the effects from interventions may spill over the adjacent communities from the intervened area and these can be examined longitudinally. After being proven in these experimental contexts, restoration of interpersonal trust could be considered to promote public health.

Several interventions could raise levels of interpersonal trust. First, possible intervention may be the more widespread participation in civil society organizations; for instance, sports clubs, social clubs, geriatric clubs, volunteer organizations, or advocacy organizations. Secondly, redesign of our public structures may also be effective to provide pleasant public spaces for better social engagements, including trees, parks, a community hall, a public house, a dance hall, or a meeting house. Third, it might help to encourage the mass media focus more on role models of trustful people. Fourth, in schools and social societies, people could learn good social skills for enhancing trust. Fifth, getting rewards for verbal and social achievements would recognize and promote their use and excellence. Sixth, public policy measures, such as prohibition of inadequate gambling or usurious lending in communities, could be instituted to prevent collapse of social cohesion. In addition, the measures that promote interpersonal trust might be different by country and by cultures and thus studies comparing differences across cultures and country boundaries would be also needed.

Our results are based on a multivariable model adjusted for potential confounders, such as demographic and socioeconomic status. In evaluating trust and QOL, we believe that these factors should be adjusted for. Individuals with higher socioeconomic status may perceive their societies as less hostile, more friendly, and have greater trust, compared with those with lower socioeconomic status [11]. At the same time, socioeconomic status is also known to be related to health status [28], [29], [30], [31]. Thus, factors of socioeconomic status may confound the observed association between trust and unhappiness. Thus, our results based on the adjusted model can be considered reliable for estimating the association between interpersonal trust and QOL.

We were also able to examine the relationships between sociodemographic factors and QOL. The significant factors for better QOL included gender, age, area size of residence, and income. The better social QOL among women compared with men in this study was consistent with previous studies [32]. Thus, the results of our study, showing poor QOL in physical, psychological, and social domains, confirmed previous reports that found poor health in people with mid-life age of 50–59 years [33], [34]. The influence of mid-life age on environmental QOL was not statistically significant but shows a pattern similar to other QOL domains in terms of the effect size of beta coefficients of age classes for this domain.

The greater psychological and environmental QOL among those living in moderate-size cities may reflect the two benefits of these cities. First, these cities may have better living conditions, such as less air and water pollution, noise, traffic volume and living cost, compared with bigger cities [35]. Second, these cities are likely to have better access to social and commercial services, such as more places for exercise, public transportation, education, art & entertainment, and shopping malls or stores [35].

The greater QOL in physical, psychological and environmental domains among the high-income group is consistent with recent surveys of Japanese adults showing that high income is associated with greater QOL [36], [37], as well as a recent European study involving seven countries, in which a higher income was associated with greater self-rated health throughout these countries [38].

Several mechanisms may explain the association between high income and greater QOL. First, high-income people may be less likely to engage in high risk behaviors, including smoking, alcohol dependence, pathological gambling, drunken or reckless driving, and commercial sexual contacts [39], [40]. Second, high-income people may be more likely to participate in regular health check-ups and to receive health-related educational opportunities [41]. Third, a higher-wage job may be associated with greater job control and less job demand with less stress [42], [43]. Therefore, in considering significant covariates for QOL, the typical “healthy” Japanese may be a woman aged 20–39 or 50–59 years, living in a moderate-size city, with high-income and greater interpersonal trust.

Interpersonal trust may induce better QOL through multiple mechanisms. First, the higher levels of interpersonal trust are related to stronger ties to friends, family and society and the increased perception of social support [44]. Consequently, having more social ties and networks leads to an individual's sense of greater well-being [45], [46]. Among the critical components for sense of well-being, including pleasure, engagement (the depth of involvement with others), and meaning (using personal strengths to serve a larger end), engagement is now considered as the most important determinant [47].

Second, interpersonal trust can lead to greater overall health in neighbors and communities and thus to more effective support and many more sources of mutual respect [28]. Third, based on the theory of the diffusion of innovations, innovative ideas diffuse more rapidly when people trust each other [48]. Rapid diffusion of valued healthy ideas may make people healthier. Conversely, the diffusion of innovation is likely to be stagnant in societies with mistrust and thus people may easily miss the opportunity to enhance health in such societies. Finally, a neighborhood rich in interpersonal trust has access to local services and amenities, and local activity groups lobbying for the provision of services are available to make a difference in terms of access to such resources [28].

Because of the analysis of cross-sectional data, our study has inferential limitations. Studies relying on instrumental variables may possibly be one of alternative procedures for correcting the endogeneity of trust and thus indicating direction of the causality. Sir Austin Bradford Hill provided nine considerations for assessing whether an observed association involved a causal component or not, including strength of association, consistency, specificity, temporality, biological gradient, plausibility, coherence, experiment, and analogy [49]. Thus, we need further research, especially an experimental study based on longitudinal data, to consolidate our finding by accumulating evidence for its causality.

Therefore, different interpretations might have been possible for our findings. For instance, self-reported QOL may be the cause of greater interpersonal trust, rather than the other way around as suggested above. It may be possible that poor QOL, particularly psychological and social QOL, may lead to social isolation and mistrust. Moreover, a third unknown and unmeasured factor could have caused higher levels of both interpersonal trust and QOL. For example, good health and greater trust may reflect different facets of an unmeasured underlying construct, such as better mental component of general well-being. However, evidence has now accumulated, indicating that psychosocial attitudes are also critical determinants for general well-being [50]. Since interpersonal trust is one of the positive psychosocial attitudes, the link between interpersonal trust and health could be understood in this context.

We conclude that interpersonal trust is associated with better QOL among Japanese adults. Further research may be needed to confirm and generalize this finding among people in other countries. Although it may be difficult to improve interpersonal trust in individual adults, there are potential measures to enhance the collective characteristics of interpersonal trust in societies. In particular, resources and investment may be needed in implementation for promoting interpersonal trust in the context of a community-based randomized interventional study. In this context, an important task for future investigations would be to identify the characteristics of civic associations and public policies that are more likely to serve the common interests and therefore improve interpersonal trust.

## Supporting Information

Appendix S1 (0.05 MB DOC)
