# Supplementary material for: Interpersonal Trust and Quality-of-Life: A Cross-Sectional Study in Japan
Source: PLoS One. 2008 Dec 19;3(12):e3985. doi: 10.1371/journal.pone.0003985 (PMC2600613; doi:10.1371/journal.pone.0003985)
Supplement: Appendix S1 — (0.05 MB DOC) [file pone.0003985.s001.doc]

**Appendix:** WHOQOL-BREF

[Questionnaire]

The following questions ask how you feel about your quality of life. I will read out each question to you, along with the response options. Please choose the answer that appears most appropriate. If you are unsure about which response to give to a question, the first response you think of is often the best one (The numbers after responses indicates the scores of the responses).

Please keep in mind your standards, hopes, pleasures and concerns. We ask that you think about your life in the last four weeks (The overall quality of life and general health facet).

1. How would you rate your quality of life?

Very poor: 1

Poor: 2

Neither poor nor good: 3

Good: 4

Very good: 5

2. How satisfied are you with your health?

Very dissatisfied: 1

Dissatisfied: 2

Neither satisfied nor dissatisfied: 3

Satisfied: 4

Very satisfied: 5

The following questions ask about how much you have experienced certain things in the last four weeks.

3. To what extent do you feel that physical pain prevents you from doing what you need to do?

Not at all: 5

A little: 4

A moderate amount: 3

Very much: 2

An extreme amount: 1

4. How much do you need any medical treatment to function in your daily life?

Not at all: 5

A little: 4

A moderate amount: 3

Very much: 2

An extreme amount: 1

5. How much do you enjoy life?

Not at all: 5

A little: 4

A moderate amount: 3

Very much: 2

An extreme amount: 1

6. To what extent do you feel your life to be meaningful?

Not at all: 5

A little: 4

A moderate amount: 3

Very much: 2

An extreme amount: 1

7. How well are you able to concentrate?

Not at all: 1

A little: 2

A moderate amount: 3

Very much: 4

Extremely: 5

8. How safe do you feel in your daily life?

Not at all: 1

A little: 2

A moderate amount: 3

Very much: 4

Extremely: 5

9. How healthy is your physical environment?

Not at all: 1

A little: 2

A moderate amount: 3

Very much: 4

Extremely: 5

The following questions ask about how completely you experience or were able to do certain things in the last four weeks.

10. Do you have enough energy for everyday life?

Not at all: 1

A little: 2

Moderately: 3

Mostly: 4

Completely: 5

11. Are you able to accept your bodily appearance?

Not at all: 1

A little: 2

Moderately: 3

Mostly: 4

Completely: 5

12. Have you enough money to meet your needs?

Not at all: 1

A little: 2

Moderately: 3

Mostly: 4

Completely: 5

13. How available to you is the information that you need in your day-to-day life?

Not at all: 1

A little: 2

Moderately: 3

Mostly: 4

Completely: 5

14. To what extent do you have the opportunity for leisure activities?

Not at all: 1

A little: 2

Moderately: 3

Mostly: 4

Completely: 5

15. How well are you able to get around?

Very poor: 1

Poor: 2

Neither poor nor good: 3

Good: 4

Very good: 5

16. How satisfied are you with your sleep?

Very dissatisfied: 1

Dissatisfied: 2

Neither satisfied nor dissatisfied: 3

Satisfied: 4

Very satisfied: 5

17. How satisfied are you with your ability to perform your daily living activities?

Very dissatisfied: 1

Dissatisfied: 2

Neither satisfied nor dissatisfied: 3

Satisfied: 4

Very satisfied: 5

18. How satisfied are you with your capacity for work?

Very dissatisfied: 1

Dissatisfied: 2

Neither satisfied nor dissatisfied: 3

Satisfied: 4

Very satisfied: 5

19. How satisfied are you with yourself?

Very dissatisfied: 1

Dissatisfied: 2

Neither satisfied nor dissatisfied: 3

Satisfied: 4

Very satisfied: 5

20. How satisfied are you with your personal relationships?

Very dissatisfied: 1

Dissatisfied: 2

Neither satisfied nor dissatisfied: 3

Satisfied: 4

Very satisfied: 5

21. How satisfied are you with the support you get from your friends?

Very dissatisfied: 1

Dissatisfied: 2

Neither satisfied nor dissatisfied: 3

Satisfied: 4

Very satisfied: 5

22. How satisfied are you with the conditions of your living place?

Very dissatisfied: 1

Dissatisfied: 2

Neither satisfied nor dissatisfied: 3

Satisfied: 4

Very satisfied: 5

23. How satisfied are you with your access to health services?

Very dissatisfied: 1

Dissatisfied: 2

Neither satisfied nor dissatisfied: 3

Satisfied: 4

Very satisfied: 5

24. How satisfied are you with your transport?

Very dissatisfied: 1

Dissatisfied: 2

Neither satisfied nor dissatisfied: 3

Satisfied: 4

Very satisfied: 5

The following question refers to how often you have felt or experienced certain things in the last four weeks.

25. How often do you have negative feelings such as blue mood, despair, anxiety, depression?

Never: 5

Seldom: 4

Quite often: 3

Very often: 2

Always: 1

[Scoring method]

Equations for computing domain raw scores:

Domain 1 (physical) score = Q3 + Q4 + Q10 + Q15 + Q16 + Q17 + Q18

Domain 2 (psychological) score = Q5 + Q6 + Q7 + Q11 + Q19 + Q25

Domain 3 (social) score =Q20 + Q21

Domain 4 (environmental) score = Q8 + Q9 + Q12 + Q13 + Q14 + Q22 + Q23 + Q24

Transformed scores were estimated using the following tables for standardizing scores from 0-100 with the lowest score of zero and the highest score of 100. (See Reference 20 for additional information)
